# Wind-Powered Wheel Locomotion, Initiated by Leaping Somersaults, in Larvae of the Southeastern Beach Tiger Beetle (*Cicindela dorsalis media*)

**DOI:** 10.1371/journal.pone.0017746

**Published:** 2011-03-23

**Authors:** Alan Harvey, Sarah Zukoff

**Affiliations:** 1 Department of Biology, Georgia Southern University, Statesboro, Georgia, United States of America; 2 Division of Plant Sciences, University of Missouri, Columbia, Missouri, United States of America; University of Hull, United Kingdom

## Abstract

Rapid movement is challenging for elongate, soft-bodied animals with short or no legs. Leaping is known for only a few animals with this “worm-like” morphology. Wheel locomotion, in which the animal's entire body rolls forward along a central axis, has been reported for only a handful of animals worldwide. Here we present the first documented case of wind-powered wheel locomotion, in larvae of the coastal tiger beetle *Cicindela dorsalis media*. When removed from their shallow burrows, larvae easily can be induced to enter a behavioral sequence that starts with leaping; while airborne, larvae loop their body into a rotating wheel and usually either “hit the ground rolling” or leap again. The direction larvae wheel is closely related to the direction in which winds are blowing; thus, all our larvae wheeled up-slope, as winds at our study site consistently blew from sea to land. Stronger winds increased both the proportion of larvae wheeling, and the distance traveled, exceeding 60 m in some cases. In addition, the proportion of larvae that wheel and the distance traveled by wheeling larvae are significantly greater on smooth sandy beaches than on beach surfaces made rough and irregular by pedestrian, equestrian, and vehicular traffic. Like other coastal species of tiger beetles, *C. dorsalis media* has suffered major declines in recent years that are clearly correlated with increased human impacts. The present study suggests that the negative effects of beach traffic may be indirect, preventing larvae from escaping from predators using wheel locomotion by disrupting the flat, hard surface necessary for efficient wheeling.

## Introduction

To move rapidly on land, animals need to generate and release sufficient energy while minimizing the friction generated as their moving body contacts the substrate. This is a formidable challenge for most crawling terrestrial animals, particularly for those short-legged crawlers with an elongate, flexible body [1], [2]. Leaping can propel animals for short distances at relatively high speeds, but among animals with worm-like builds, it has been reported to date only for the larvae of a number of fly species [3], [4], [5], and for juveniles of certain entomophagous nematodes [6], [7].

Wheel locomotion is a means by which animals can cover larger distances quickly. In wheel locomotion, the animal's entire body rotates forward around a single axis, propelled by either the passive force of gravity or the active force of the animal's movements.

Although wheel locomotion is one of the most efficient forms of travel, it also faces significant limitations that probably account for its near-absence in nature. Wheels lose most of their efficiency in any but smooth, hard surfaces, have difficulty clearing all but the lowest obstacles, and are less maneuverable than other forms of locomotion [8], [9]. Few environments meet the first two conditions, and few motives for efficient locomotion are free from the need for maneuverability [10].

Gravity-driven wheel locomotion is known in the wheel spiders (*Carparachne* spp.), which live on the steep sand dunes of the Namib Desert in Africa [11]; these spiders cartwheel; that is, their body is centered on the axis of rotation, with their legs projecting at angles to form spokes and bent distally to form a rim. Active wheel locomotion has been documented in the tropical American mantis shrimp *Nannosquilla decemspinosa* and in caterpillars of the moths *Pleurotya ruralis* and *Cacoecimorpha pronubana* [12], [13]. These species have elongate, flexible, short-legged bodies; they form wheels by coiling their bodies, dorsal-side-out, bringing the head and tail ends together and then actively rolling themselves along the substrate.

Adult tiger beetles are among the fastest running insects, but larvae are soft-bodied, sedentary burrow-dwellers that essentially never leave their burrows until they emerge as adults [14]. Rapid locomotion would seem to be irrelevant for a fossorial lifestyle and difficult to achieve with a “worm-like” morphology; nonetheless, we discovered that larvae of the southeastern beach tiger beetle, *Cicindela dorsalis media*, were able to travel considerable distances quickly. In this study, we documented the mechanisms and performance capacity of this mode of locomotion, and investigated the effects of surface irregularities in beach topography caused by pedestrian and vehicular traffic.

## Methods

### Study sites

We conducted field studies during the summer of 2007 and then again in the summer of 2010. Our study area was the southeastern shore of Cumberland Island National Seashore (CINS), St. Mary's, Georgia, USA. Cumberland Island, the largest barrier island off of Georgia's coast, is characterized by shallow sloping beaches with prevailing winds from the east and northeast in spring (March – May) and from the north in summer (June – July) [15]. Although most of CINS is federally protected, there is still considerable disturbance on the beach, primarily due to human recreational activity and feral animals. CINS is a popular tourist destination with 45,000 visitors annually. A major attraction of the island is a population of about 200 feral horses that frequently roam the beach. There are also 200–300 feral hogs that may visit the beach during turtle nesting season. Vehicle traffic on the beach is limited to residents and park staff, but plans for vehicle tours on the north side of the island were approved in 2009 [16].

### Study organism

The southeastern beach tiger beetle, *Cicindela dorsalis media*, is found along the Atlantic Coast from New Jersey to Florida on wide sandy beaches [17]. Although its numbers have been declining precipitously over the last couple of decades [18], [19], [20], it is considerably more common than its federally endangered conspecific *C. d. dorsalis* [21]. Whereas *C. dorsalis* larvae have been reported to be found between the upper intertidal and the low dunes and to be primarily nocturnal [17], we have found that *C. d. media* larvae at CINS are found primarily in the mid to upper intertidal zone, and are as active by day as by night (pers. observ.).

We located larvae of *Cicindela dorsalis media* on the beaches by surveying the mid to upper intertidal zone for burrows visually. Larvae were collected one at a time by approaching a larva at the mouth of its burrow carefully, plunging a trowel suddenly into the soil in front of the burrow at a roughly 45° angle to block the larva's retreat, and then lifting the entire clump of sand out of the ground. Each larva was used promptly and in only a single trial, and then returned unharmed to the sand. Some larvae were videotaped at normal speed using a Sony DCR-TRV22 digital camcorder (2007) or at 300 fps using a Casio Exilim EX-F1 (2010).

### Behavioral observations

Our basic approach was to place a larva on the surface of the sand and probe it gently with a thin twig or grass blade for one minute or until the larva exhibited wheeling behavior. We qualitatively recorded the response of the larva to the probing (i.e., death feign, crawl, leap). For leaping larvae, we recorded whether they also wheeled. For wheeling larvae, we recorded the distance and direction traveled; in 2007, we recorded this data for leaping larvae as well. In 2007 we estimated wind direction with a compass and windsock, and measured wind speed with a Kestrel 3000 Pocket Weather Meter. In 2010 we used a vane-mounted Kestrel 4500 Pocket Weather Tracker to more precisely record wind speed and direction. We recorded head capsule width as an indicator of larval size (in 2007 we also recorded total body length). Trials were conducted on a smooth section of sand in the midst of the larval burrow zone.

In 2010, we examined the effect of beach surface roughness on wheeling behavior. To determine whether beach surface regularity affected the ability of larvae to initiate and maintain wheeling, we conducted the above tests in two adjacent locations on the beach: a smooth, intertidal section and an adjacent, higher region where the surface was much more uneven due to considerable human, vehicular, and horse traffic. To minimize potential confounding effects of changing winds or temperatures during the course of a day's trials, we alternated between smooth and rough sites after every three to five tests.

### Data analysis

We used simple χ^2^ continency tests to assess the effect of beach substrate on the likelihood that a larva would leap during a trial (Fig. 5A), and to assess the effect of beach substrate on the likelihood that a leaping larva would subsequently wheel (Fig. 5B). We use nominal logistic regression to assess the effects of wind speed and beach roughness on the probability that leaping larvae would wheel (Table 2; Fig. 4). Wheeling distances were not normally distributed (2007 data: Shapiro-Wilk W = 0.48, p<0.001; 2010 data: W = 0.51, p<0.001), and so were log-transformed for statistical analyses (2007 data: W = 0.98, p = 0.15; 2010 data: W = 0.97, p = 0.07). We used separate slopes analysis of covariance, with wind speed as the covariate, to assess the effect of larval behavior (i.e., leaping vs. wheeling) on distance traveled (Table 1, Fig. 3) and the effects of beach roughness on distance wheeled (Table 3, Fig. 6) and direction wheeled (Table 4, Fig. 8). We used correlation analysis to assess the relation ship between larval length and head capsule width.

**Table 1:** Effects of larval behavior and wind speed on distance traveled by *C. dorsalis media* larvae.

Source	DF	F	*p*
Behavior	1	102.40	<.0001***
Wind speed	1	22.13	<.0001***
Behavior*wind speed	1	10.54	0.0015**

Note: *Behavior* refers to whether or not larva wheeled in response to prodding. Distance traveled log-transformed for analysis. 2007 data on smooth surfaces.

**Table 2:** Effects of beach roughness and wind speed on proportion of *C. dorsalis media* larvae that wheeled.

Source	DF	χ2	p
Roughness	1	27.27	<.0001***
Wind speed	1	5.00	0.025*
Roughness*wind speed	1	1.35	0.244 N.S.

Note: *Roughness* refers to substrate surface (smooth vs. roughened by prior foot or vehicular traffic).

**Table 3:** Effects of beach surface properties and wind speed on distance traveled by *C. dorsalis media* larvae.

Source	DF	F	*p*
Roughness	1	5.74	0.008**
Wind speed	1	0.97	0.47 N.S.
Roughness*Wind speed	1	4.89	0.13 N.S.

Note: *Roughness* refers to substrate surface (smooth vs. roughened by prior foot or vehicular traffic). Distance traveled log-transformed for analysis.

**Table 4:** Effects of beach roughness and wind direction on direction wheeled by *C. dorsalis media* larvae.

Source	DF	F	*p*
Roughness	1	0.18	0.677 N.S.
Wind direction	1	25.00	<.0001***
Roughness*Wind direction	1	1.39	0.248 N.S.

Note: *Roughness* refers to substrate surface (smooth vs. roughened by prior foot or vehicular traffic).

## Results

### Behavioral observations/description of wheeling

Larval tiger beetles unambiguously use wheel locomotion as a response to disturbance. Without benefit of high-speed video, however, a typical wheeling event looks like a brief and violent bout of thrashing on the sand, interspersed with an occasional leap, after which the larva suddenly and rapidly zips along the surface of the sand in a more or less straight line (Video S1). Most of the detailed descriptions that follow were obtained through careful frame-by-frame inspection of 48 high-speed video clips.

When a larva is touched on the head, thorax, or anterior abdomen, it typically jerks or crawls away, threatens with open jaws without arching backwards, contracts its body into a sinuate death-feigning pose, or regurgitates. Death-feigning and regurgitation are known in adult tiger beetles [14], but to our knowledge have not been previously reported in larvae.

**Figure 1:**
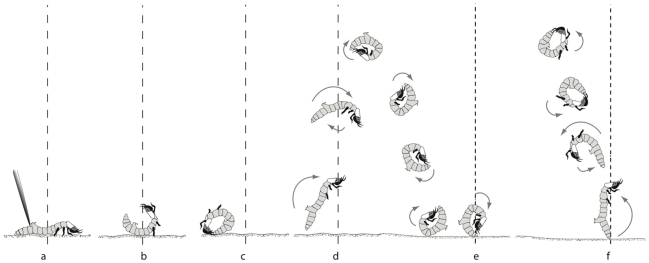
A *generalized* leaping and wheel initiation sequence in *C. dorsalis media* larvae. The number, height, and direction of individual components vary markedly depending on wind speed and fine-scale surface topography. The two dotted lines (coarse and fine) calibrate the successive illustrations in two locations as the larva moves along the beach. Sequence of events: a) being prodded; b–c) coiling and rolling backwards until pygopod (tail) contacts sand; d) straightening out, pushing off sand into the air, rotating forward; e) landing on sand; at this point larva either begins wheeling, returns to extended position (a), or proceeds to f) when pygopod or head contacts sand, straightening out, pushing off sand into the air, rotating backward; when larva lands on sand, proceeds to either (a) or (c).

However, when a larva is touched on the posterior part of the abdomen (i.e., from the fifth abdominal segment to the tail), it vigorously arches its body backwards so that its head snaps upwards and backwards and its tail (if not pinned to the substrate) arches upwards and forwards (Fig. 1a–b). Although this motion brings the head with its open mandibles towards the disturbing agent (in our case the poking twig), larvae usually did not bite the stick. Rather, the larva arches its head over and past its abdomen until its head and tail ends meet, resulting in the animal's body forming a “ventral side out” loop. It is not clear whether the animal holds the head and tail ends together: sometimes the two ends seem in direct contact, sometimes the head end contacts the abdomen between the tail and the fifth abdominal segment, sometimes the tail contacts the thoracic region posterior to the head capsule. The momentum of the head end coiling backwards causes the entire animal to roll backwards until the tail of the now-coiled animal contacts the substrate (Fig. 1c).

As soon as its tail contacts the sand, the larva attempts to launch itself off the sand by arching its body suddenly in the opposite direction, using its tail as an anchor. The larva now forms a dorsal-side-out loop that rotates forward while in the air, often completing one to several rotations while airborne (Fig. 1d; Video S2). When a larva lands on the sand, it typically will either fall over on its side or else start to roll. In the latter case it will either continue to wheel or else relaunch (Fig. 1f) once its tail (or less commonly head) contacts the sand (Fig. 1e). In this fashion larvae rapidly and repeatedly alternate between dorsal-side-out and ventral-side-out positions up to five times after a single poke before either wheeling or ceasing activity (Video S3). The specific trajectory of each leap was clearly influenced by the velocity of the rolling larvae and that of the wind. In addition, slight variations in the timing of the leap during a roll, as well as fine-scale surface irregularities, had apparent but difficult-to-quantify effects on leap trajectories. The net result is that in the absence of high-speed video, larvae appear to be wildly flipping and thrashing on the surface.

Larvae were occasionally observed to wheel on the substrate briefly in a ventral-side-out loop, but only in the dorsal-side-out position would larvae wheel more than one or two revolutions. Larvae are equally likely to wheel headfirst or tail-first.

While wheeling, larvae normally maintained a rounded loop. On smooth sand, their body was more or less continuously in contact with the sand while rolling, but mild irregularities or low sand ridges caused larvae to bounce instead of roll along the shore. Bouncing wheels appeared to be increasingly common with increasing wind speed, although we did not measure this relationship. This irregularity-induced bouncing appeared to be beyond larval control, although we saw no indication that bouncing wheels were less effective than rolling wheels. Larvae would sometimes actively maintain wheeling runs by pushing off the sand with their tail while rolling. This produced a low, bouncing leap that continued to propel the larva forward, usually in the same direction it had been wheeling. However, sometimes larvae that were obviously decelerating when they pushed off were seen to change direction by up to 90° (though still heading up-slope) (Video S4).

During the entire process of leaping and wheeling, the three pairs of legs extend outward nearly perpendicular to the body plane; the legs appear to help the larva maintain balance while wheeling, based on several high-speed video clips in which a wheeling larva wobbled slightly from side to side without falling over.

Wheeling bouts ended in one of three ways. Occasionally the larva would unwind from the loop while still wheeling, which immediately ended the bout. More typically, wheeling velocity would slow to the point that the larva would fall over on its side still looped. At our study site, the most common cause for such reduced velocities was an encounter with a mild obstacle, such as a low sand ridge, loose clump of sand, or rough patches caused by footsteps or hoof prints. Sometimes these obstacles were severe enough to bring wheeling to an immediate halt. In any case, once a larva stopped wheeling, it completely uncoiled, dorsal-side-up, and began to either crawl away or else burrow into the sand, sometimes after a brief pause (Video S4).

**Figure 2:**
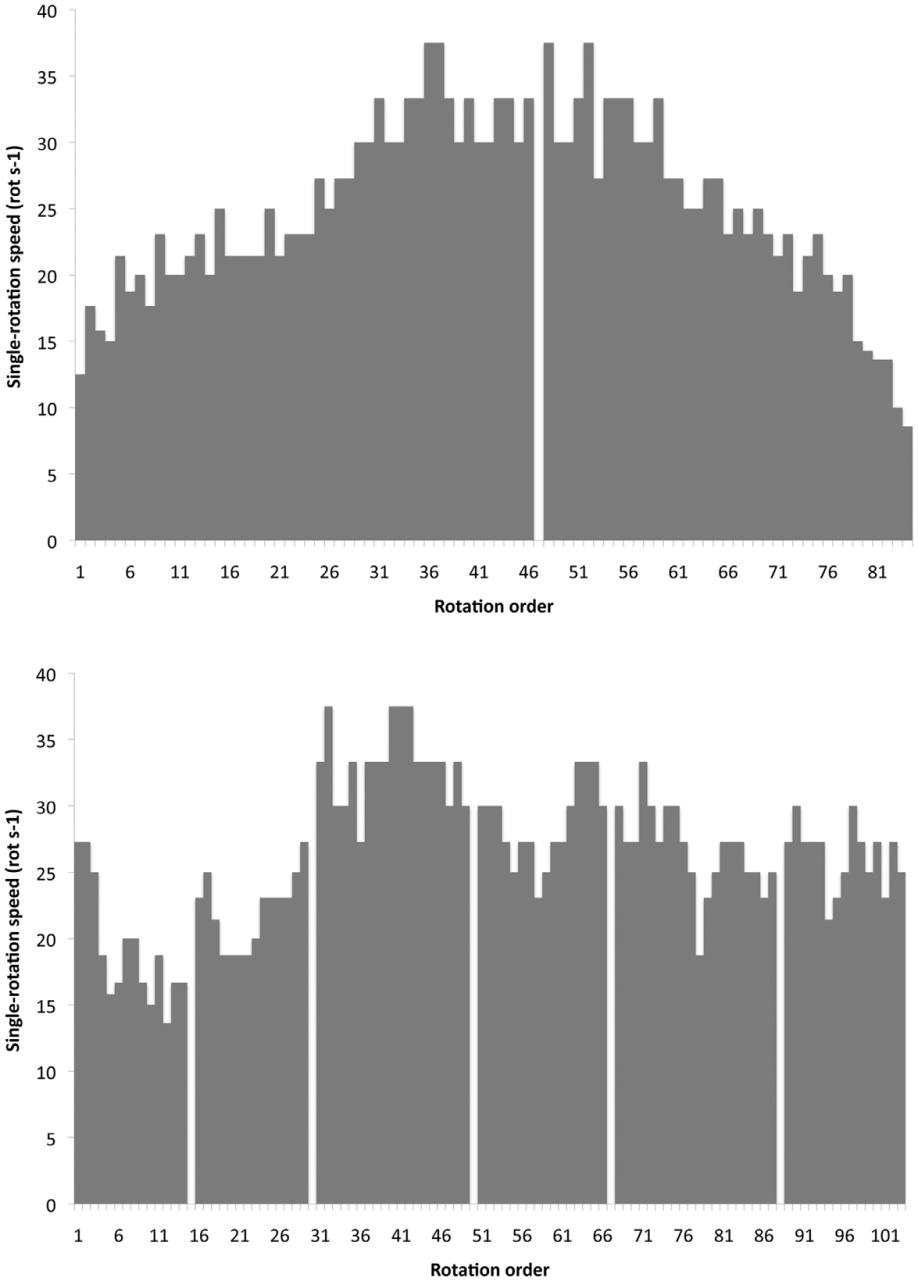
Instantaneous rotational speed of two wheeling *C. dorsalis media* larvae during separate wheeling events. Each bar represents a single complete rotation. Missing values indicate intervals of varying length during which the video camera momentarily lost track of the larva (see, e.g., Video S4).

Wheeling speeds and rotation rates are clearly dependent on fine scale changes in wind speed and surface topology during a run, rendering mean values of questionable utility (Fig. 2). Winds at the time of the video trials ranged from approximately 3.5–5.5 m/s. During most runs larvae maintained rotational rates of 20–30 Hz (hertz, or rotations per second), peaking at 37.5 Hz. For our video trials we recorded larvae that were approximately 15 mm long. This translates to a typical speed of 0.30–0.56 m/s), assuming a larva travels one body length per rotation. Under the high winds of 2007 (>12.5 m/s), we observed larvae that were wheeling faster than our assistant could run on the beach, which we calculated separately to be around 3 m/s.

**Figure 3:**
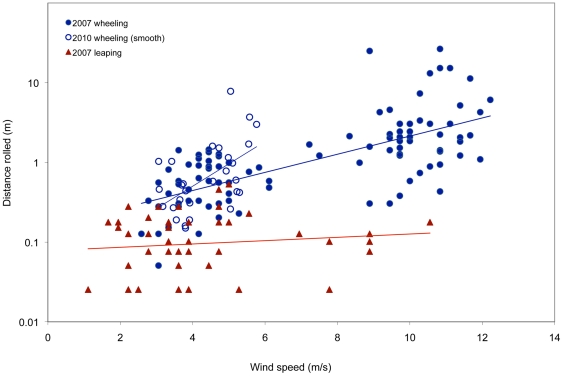
Effect of wind speed on distance traveled by leaping and wheeling *C. dorsalis media* larvae. These trials were done on smooth beach surfaces, including all data from 2007 and from the “smooth” treatment in 2010.

In 2010, larvae wheeled distances of up to 10 m (Fig. 3). In the stronger winds of 2007, larvae wheeled up to 25 m during our experimental trials (Fig. 3), and we incidentally recorded much longer events, one exceeding 60 m. Larvae often did not wheel in a straight line, but we did not record the frequency, extent, or direction of this curving; thus, our straight-line measurements tend to underestimate slightly the actual distance traveled by larvae.

**Figure 4:**
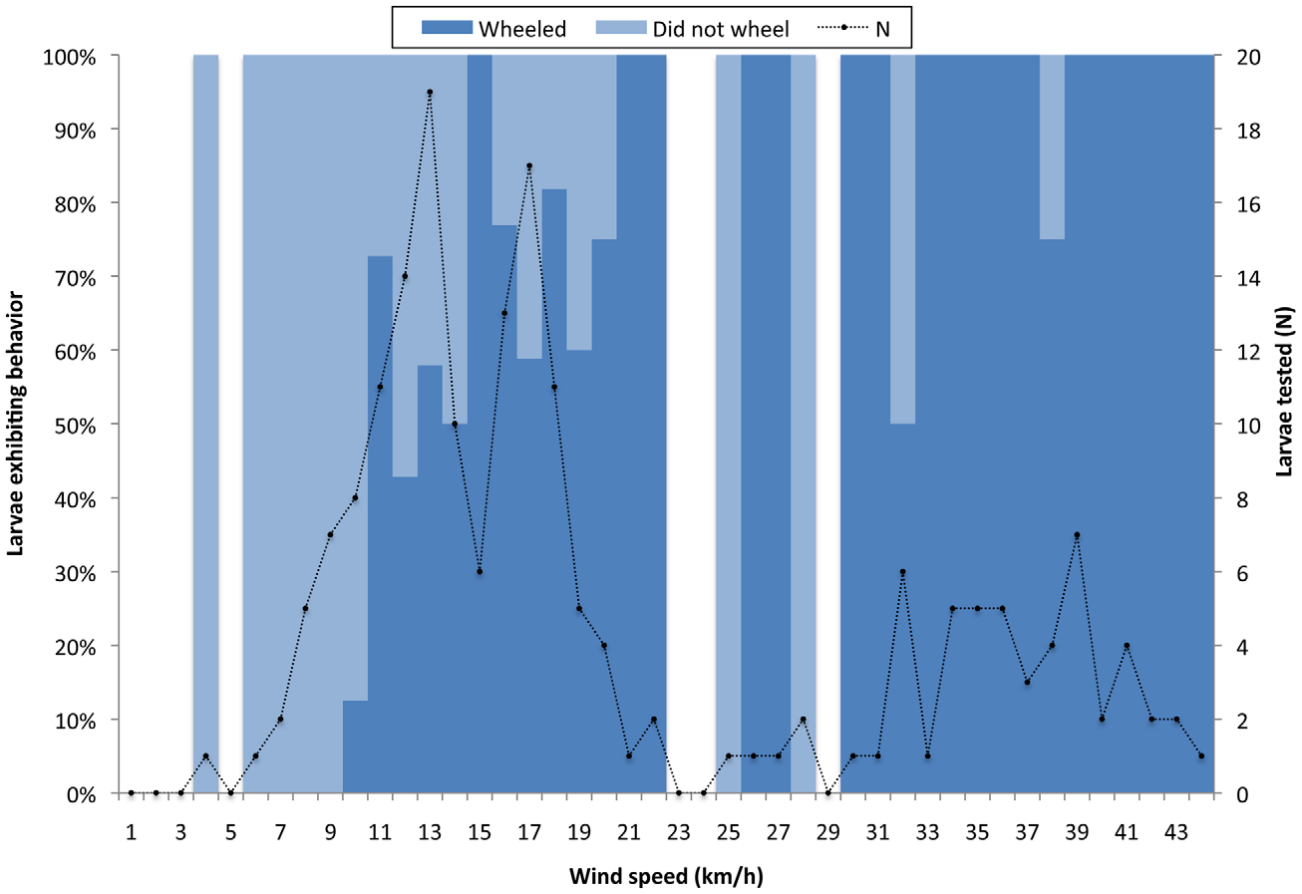
Effect of wind speed on probability that leaping *C. dorsalis media* larvae would wheel. These trials were done on smooth beach surfaces, including all data from 2007 and from the “smooth” treatment in 2010.

On average, wheeling larvae moved significantly further from their starting point (234.32±438.06 cm, or 147.95±284.16 body lengths; N = 46) than did leaping-only larvae (13.20±10.93 cm, or 8.40±6.41 body lengths; N = 99) from their starting point (Fig. 3, Table 1). The proportion of leaping larvae that wheeled increased significantly with wind speeds (χ^2^ = 20.79, p<0.001, N = 140; Fig. 4), as did the distance traveled by wheeling larvae (R^2^ = 0.47, p<0.001; Fig. 3; Table 1). However, the distance traveled by leaping-only larvae was not affected by wind speed (Fig. 3; interaction effect in Table 1; for leaping-only larvae, R^2^ = 0.01, p = 0.42).

### Effects of fine-scale substrate topology

**Figure 5:**
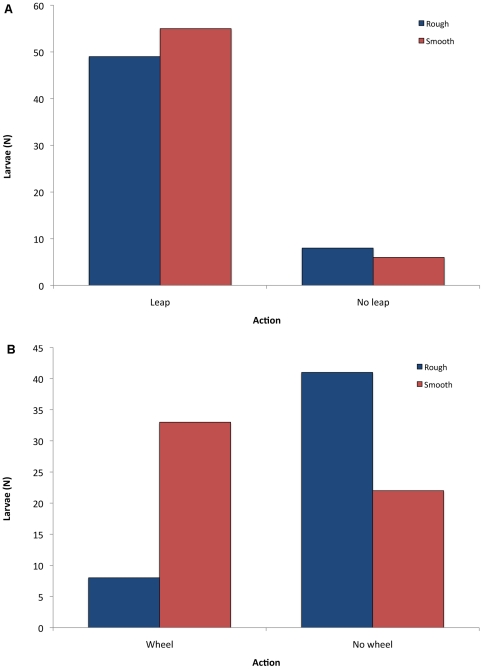
Effect of beach roughness on rapid locomotion in *C. dorsalis media* larvae. **A**. Number of larvae that leaped when tested on smooth vs. rough beach surfaces. **B**. Number of leaping larvae that wheeled when tested on smooth vs. rough beach surfaces.

In 2010, we tested 118 larvae (61 on smooth substrate, 57 on rough substrate). Overall, nearly 90% of the larvae leaped within the first minute; the remainder feigned death for the full minute. The roughness of the surface did not affect whether or not larvae leaped (χ^2^ = 0.50, *p* = 0.48; Fig. 5A). Leaping larvae were significantly more likely to wheel on smooth sand than on rough sand (60% vs. 16.3%; χ^2^ = 20.51, *p*<<0.001; Fig. 5B; Table 2; Video S3); only eight of 49 leaping larvae wheeled on rough surfaces.

**Figure 6:**
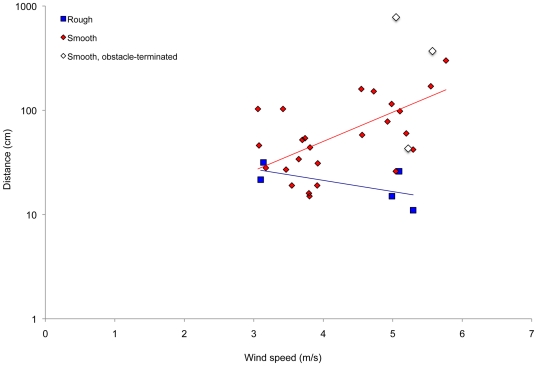
Effect of wind speed and beach roughness on distances wheeled by *C. dorsalis media* larvae. Open diamonds represent larvae on smooth substrate whose wheeling bouts were truncated by encounters with isolated obstacles.

Wind speeds were more limited during our experiments in 2010, ranging between 2.4 and 5.8 m/s. Unfortunately, we lost weather data from the first day of these trials, meaning that we had wind speed data for only 30 of our 41wheeling larvae (5 of 8 from rough sand and 25 of our 33 from smooth sand). Three wheeling events on smooth sand were prematurely terminated when a larva rolled into an obstacle (e.g., a rough patch of sand or sand ridge; Fig. 6). These larvae were excluded from distance-related calculations because they underestimate the distance the larvae would have travelled had the sand stayed smooth. As in 2007, the proportion of leaping larvae that wheeled increased with wind speed (Table 2), with no significant interaction with beach roughness. When beach roughness and wind speed are analyzed together in a separate slopes ANCOVA, wheeling larvae traveled significantly farther on smooth sand than on rough sand (Table 3, Fig. 6), even excluding the obstacle-terminated wheeling runs, which serve to illustrate directly the consequences of beach surface complexity on the wheeling ability of larvae. However, in this analysis, wind speed does not affect the distance larvae wheel, which may reflect the smaller sample sizes or more limited range of wind speeds sampled in 2010 vs. 2007.

### Direction wheeled vs. direction of wind

**Figure 7:**
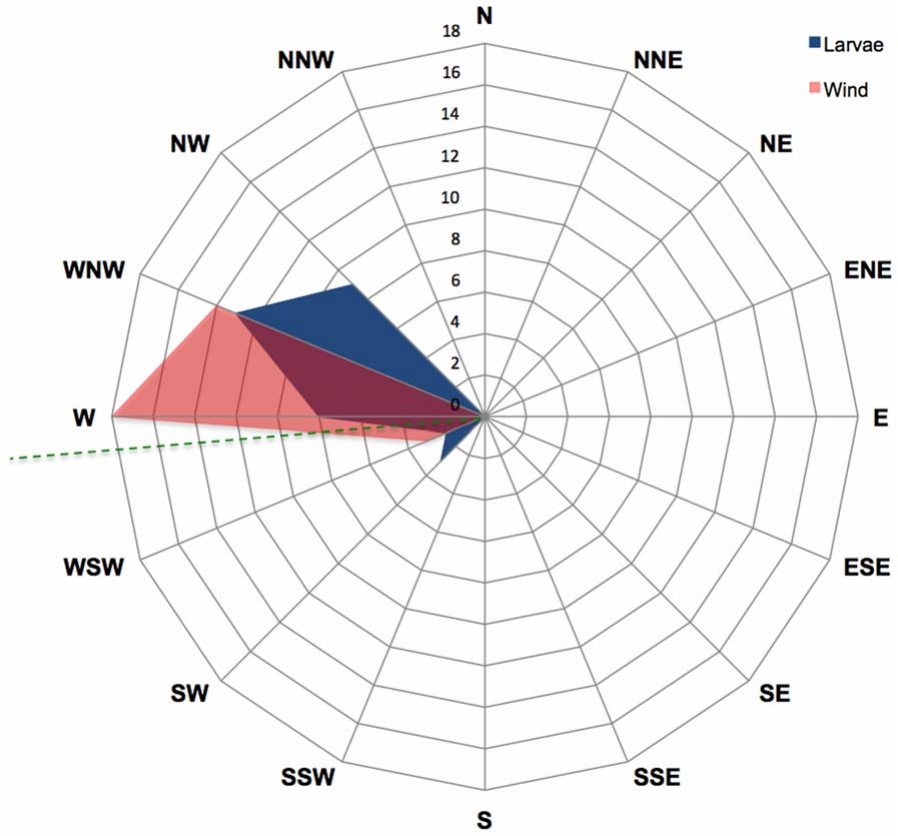
Heading of *C. dorsalis media* larvae and of winds during wheeling events. The dashed line represents the direction of steepest slope up the beach at the study site. Data shown for larvae tested on smooth surfaces (N = 35).

**Figure 8:**
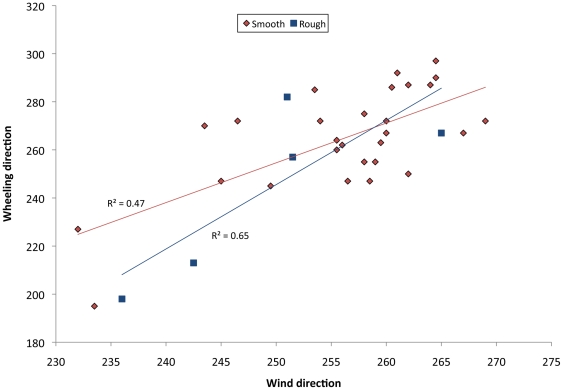
Effects of wind heading and substrate roughness on wheeling direction in *C. dorsalis media* larvae. Regression coefficients are for substrate categories analyzed separately; see text and Table 4 for combined analyses.

At the study site, the beach ran nearly, but not quite, along a due north-south axis at our study site, with the slope running directly uphill (i.e., perpendicular to shore) at 264°. Prevailing winds blew from ocean onshore; during our tests readings ranged from 232° to 269° (Fig. 7). All wheeling larvae rolled up-slope, with compass headings ranging from 195–297° (Fig. 7). There was a strong positive correlation between the direction larvae rolled and the direction the wind blew towards during the trial, which was unaffected by substrate roughness (Fig. 8; R^2^ = 0.57; Table 4).

### Body size

In 2007, head capsule width and body length were significantly correlated, but the relationship was weaker than expected (R^2^ = 0.35, p<0.001, N = 34), primarily because of the difficulty in getting precise and accurate body length measurements of live, soft-bodied larvae in the field. Thus, we measured only head capsule width in 2010. When controlling for substrate effects, body size (as estimated by head capsule width) affected neither the probability that larvae wheeled (p = 0.57), the distance (p = 0.83), nor the direction (p = 0.76) that larvae wheeled.

## Discussion

Under certain circumstances, larvae of the southeastern beach tiger beetle *Cicindela media dorsalis* use a remarkable form of wheel locomotion. Furthermore, this wheeling is preceded, and facilitated, by a series of catapulting jumps that are quite remarkable in their own right. Perhaps most surprisingly, we found this spectacular but previously unnoticed suite of behaviors in “one of the best-studied insect species in North America” [22]. Our initial discovery of this behavior was itself serendipitous: one of us (SZ) was walking through some unusually loose sandy drifts on Cumberland Island and happened to kick up some *C. d. media* larvae, which promptly started wheeling. We have not seen such drifts on Cumberland Island during subsequent visits. Larval tiger beetles are normally alert and quickly drop down into their burrows at the approach of potential predators; on subsequent visits we have been unable to dislodge larvae by kicking sand drifts, nor have we seen tiger beetles wheeling except when we provoked them ourselves. Although the discovery of leaping and wheeling was fortuitous, and the behaviors themselves apparently rarely expressed, they nonetheless are easily and consistently elicited and do not appear to be unnatural artifacts.

Leaping represents serious challenges to soft-bodied, elongate, short-legged or legless animals [2], and only a few examples have been reported previously. In each case, the animal bends its body into a tight loop by bringing its head and tail ends together, holding the ends together with a catch mechanism that, when suddenly disengaged, releases the energy stored in the tensed body loop and thus launching the animal. Fungus gnats reach backwards with their heads to form a ventral-side-out loop [3], whereas cheese flies, Mediterranean fruit flies, and nematodes form dorsal-side-out loops [4], [5], [6]. In contrast, *C. d. media* larvae are equally proficient at leaping from either position, starting from a ventral-side-out position and then typically alternating between the two in a given leaping-rolling sequence.

In nematodes, the loop is maintained by water tension from the water film covering the body of the animal, whereas the much larger fly larvae use various pegs or hooks to secure the ends of the loop. It was unexpectedly difficult to determine the precise catch mechanism in *C. d. media*. There may well not be a single mechanism, given the numerous potential gripping structures, setae, ridges, and pockets at both ends of the larvae, and the fact that they leap from both dorsal-side-out and ventral-side-out positions. It is also possible that they do not use a pure catch-and-release mechanism, gaining power by combining elastic release with real-time muscle contraction.

The function of leaping in *C. d. media* clearly differs from that in other soft-bodied leapers. Fly larvae leap to minimize predation risks while changing microhabitats prior to metamorphosis [3], [4], [5]; nematodes leap onto or towards potential hosts [23]. In *C. d. media*, one benefit of leaping is likely to be to gain some immediate separation from an attacker. In response to attacks by the tiphiid wasp *Methocha*, the larvae of several species of tiger beetles have been observed flipping out their burrow, thrashing about on the surface, and then crawling away ([24], [25]). This flipping and thrashing behavior sounds similar to the leaping that precedes wheeling in *C. d. media*.

Leaping also initiates the process of wheeling. This is particularly important for a wind-powered wheeler like *C. d. media*; self-powered and gravity-powered wheelers do not leap. Whereas a larva may be facing any direction as it leaps, wheeling requires it to be oriented along the axis of the wind. Although the process of alignment with the wind axis during a leap is apparent in our videos, the mechanisms appear to be complex and beyond the scope of this paper. Because wind speeds increase with increasing height above the surface (i.e., the wind gradient), leaping larvae get a stronger initial push by the wind than they get on the surface. Also, initiating a rotating wheel while airborne presumably encounters less frictional resistance than while on the ground. This may allow the larvae to more quickly reach rotational speeds rapid enough to maintain balance while wheeling along the surface. Certainly some of the rotational energy generated while airborne gets converted to translational energy when the larva hits the sand, propelling it forward at least briefly until the wind takes over.

Although a burrow-dwelling, elongated, soft-bodied animal might seem an unlikely candidate for either wheeling or leaping, three of the four previously known examples of wheeling are also elongate, flexible animals that live in burrows (*Nannosquilla*) or equivalent shelters (i.e., rolled leaf tubes in *Pleurotya* and *Cacoecimorpha*). Gould [26] argues that it would not be possible for animals to evolve wheeled appendages because there is no way for nutrients and nerve impulses to move between the wheel and the body of the animal. However, this does not preclude an animal from temporarily deforming its entire body to form a wheel. Such deformations would seem to be easiest for an animal whose body is either already somewhat wheel-shaped (e.g., “cartwheeling” spiders) or else elongated and flexible enough to form a head-to-tail loop.

*C. d. media* larvae, unlike other wheeling animals, uses the wind to power its wheeling. This leads to the unique result that they normally roll uphill, because at coastal locations prevailing winds typically come from offshore during the daytime. At our study site on Cumberland Island, prevailing winds blew mostly from due east, and the beach slope rose nearly due west; as a result, larvae always wheeled strongly up-slope. This eventually led them into the rougher lower dune area of the beach, which quickly terminated wheeling. However, at a location on nearby Jekyll Island that housed a large population of *C. d. media*, we observed that prevailing winds tended to run more parallel to shore, which would allow the possibility of wheeling distances much greater than the 60 m maximum we observed at the Cumberland Island site. Wind-powered wheeling could create potential problems for larvae when winds are blowing out to sea (e.g., at night), but they are able to terminate rolling actively. We have not yet determined whether larvae engage in these behaviors at night.

Not surprisingly, the velocity of wind is important in both initiating and sustaining wheeling bouts; we did not observe larvae wheeling in winds below 2.8 m/s, nor did they wheel for more than a meter in winds less than 4.2 m/s. The roughness of the substrate also strongly affected the wheeling ability of larvae, which had significantly greater success at initiating wheeling, and traveled significantly farther, on smooth sand than on roughened sand. Similarly, Schurr et al. [27] showed that for seeds of a given size, dispersal on the ground increases with wind speed and decreases with obstacle density.

It is instructive to compare the performance of wheeling *Cicindela dorsalis media* larvae to the handful of other known wheeling species (Table 5). The two passive wheelers (*C. dorsalis media* and *Carparachne aureoflava*) can achieve far greater speeds and distances than can the three self-propelled active wheelers. Of course, the ability of passive wheelers to realize this potential is completely dependent on local conditions. Thus, *C. d. media* is unable to wheel on rough substrates or when winds drop below 2.8 m/s, whereas *C. aureoflava* requires slopes greater than 15° [11]; active wheelers are not so constrained.

**Table 5:** Wheel locomotion performance across taxa.

Species	Power source	Distance (m)	Rotational rate (Hz)	Speed (m/s)
*Cicindela dorsalis media*	wind	0.5–5 (60)	20–30 (37.5)	0.30–0.56 (3.0)
*Carparachne aureoflava*	gravity	1–100	20.6±8.4	1±0.30
*Nannosquilla decemspinosa*	self	<1 (2)	?	0.015–0.045 (0.056)
*Pleurotya ruralis*	self	<0.125	16	0.40±0.04
*Cacoecimorpha pronubana*	self	?	11.4	0.29

Values are either means (±S.D. if provided) or typical ranges; maximum values in parentheses. Maximum distance and speeds for C. d. media were recorded outside experimental trials.

We have not yet established the function of wheeling in *Cicindela dorsalis media*. Other species wheel primarily to escape either suboptimal environmental conditions [28] or predators [11], [13], both of which are plausible candidates in *C. d. media*. In tiger beetles, wheeling is only an option for larvae that are not in their burrows, and tiger beetle larvae rarely leave their burrows voluntarily. Larvae of the coastal *C. hirticollis* are known to change burrow locations in response to environmental conditions [29]. *C. d. dorsalis* is also exceptional in this respect, and will move if conditions are poor [21]; furthermore, in more northern populations, the springtime burrows of first instar larvae tend to be close to the water's edge, whereas those of later instars, which appear in the fall, are much further upslope, presumably to avoid the erosion of the lower beach that results from strong waves and winds in the winter [30]. As far as is known, the larvae of these species change positions by crawling across the substrate (B. Knisley, T. Simmons, pers. comm.), but presumably could wheel if attacked by a predator during the process of relocation.

Nonetheless, we suspect that wheeling is primarily an enhanced response to attack by *Methocha*, which is considered to be one of the most important sources of larval mortality in tiger beetles [14], [25]. Other species follow *Methocha*-induced bouts of flipping and thrashing by attempting to crawl away. Sometimes this behavior is sufficient to elude the wasp, but sometimes the wasp is able to follow the larvae on the surface, and either drag them back to the original burrow or bury them on the spot [24]. Wheeling rapidly moves larvae much farther away from the burrow entrance than does leaping alone, and it seems highly unlikely that *Methocha* would be able to follow, much less keep pace with, a wheeling larva.

The wide, flat, sandy beaches favored by *C. dorsalis* are ideally suited for wheeling, and the nearly continuous sea breezes represent a reliable power source for wheeling, at least during the day. However, wheeling in *C. d. media* is severely compromised by the beach roughness introduced by levels of pedestrian, vehicular, and equine traffic that can occur at CINS. Therefore, if wheeling is a means to escape predators, then trampling will lead to increased larval mortality due to predation. The severity of this effect should increase with both predator attack rates and intensity of traffic. This may provide at least a partial explanation for the precipitous decline noted for *C. d. media* on beaches with heavy pedestrian or vehicular traffic [18].

## Supporting Information

Video S1 **Typical leaping and wheeling sequence by a** ***C. dorsalis media*** **larva.** Normal speed video taken with a consumer-grade Sony DCR-TRV22 Digital Handicam.
(MP4)

Video S2 **Single leaping somersault event by a** ***C. dorsalis media*** **larva.** 300fps (10X normal speed) taken with a Casio Exilim EX-F1 digital camera.
(MP4)

Video S3 **Sequence of leaping somersaults on rough sand by a** ***C. dorsalis media*** **larva.** 300fps (10X normal speed) taken with a Casio Exilim EX-F1 digital camera.
(MP4)

Video S4 **Leaping and wheeling sequence by a** ***C. dorsalis media*** **larva.** 300fps (10X normal speed) taken with a Casio Exilim EX-F1 digital camera. The leaping response of this larva to probing was somewhat more delayed than is typical. The video illustrates the various consequences of obstacles during a wheeling run (i.e., bouncing, deceleration, and termination of the run). It also shows the larva leaping to extend its run without further experimental contact, resulting in a considerable change of direction (though still wheeling up-slope).
(MP4)
